# On the role of phase separation in the biogenesis of membraneless compartments

**DOI:** 10.15252/embj.2021109952

**Published:** 2022-02-02

**Authors:** Andrea Musacchio

**Affiliations:** ^1^ Department of Mechanistic Cell Biology Max Planck Institute of Molecular Physiology Dortmund Germany

**Keywords:** Organelles, Structural Biology

## Abstract

Molecular mechanistic biology has ushered us into the world of life’s building blocks, revealing their interactions in macromolecular complexes and inspiring strategies for detailed functional interrogations. The biogenesis of membraneless cellular compartments, functional mesoscale subcellular locales devoid of strong internal order and delimiting membranes, is among mechanistic biology’s most demanding current challenges. A developing paradigm, biomolecular phase separation, emphasizes solvation of the building blocks through low‐affinity, weakly adhesive unspecific interactions as the driver of biogenesis of membraneless compartments. Here, I discuss the molecular underpinnings of the phase separation paradigm and demonstrate that validating its assumptions is much more challenging than hitherto appreciated. I also discuss that highly specific interactions, rather than unspecific ones, appear to be the main driver of biogenesis of subcellular compartments, while phase separation may be harnessed locally in selected instances to generate material properties tailored for specific functions, as exemplified by nucleocytoplasmic transport.

## Graphical abstract

This commentary contrasts the contributions of site‐specific versus unspecific biomolecular interactions to the molecular mechanisms of subcellular compartment assembly and function.

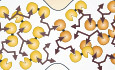

**Glossary**
CPC: chromosomal passenger complex
*C*_sat_
: saturating concentration
FRAP: fluorescence recovery after photobleaching
IDP: intrinsically disordered protein
IDR: intrinsically disordered regions
K_D_
: dissociation constant
*k*_off_
: dissociation rate constant
MVP: multivalent protein
NoLS: nucleolar localization signal
NOR: nucleolar organizer region
NPC: nuclear pore complex
NTR: nuclear transport receptor
Nups: nucleoporins
PRM: proline‐rich motif
PS: phase separation
RNP: ribonucleoprotein
SAC: spindle assembly checkpoint
SSI: site‐specific interaction

## Introduction

Many functions of eukaryotic cells are confined within compartments that are not delimited by membranes (and therefore often referred to as *membraneless*). Examples that have been known for many decades, among a number of others, are the centrosome, the nucleolus, and P‐granules (Courchaine *et al*, 2016; Banani *et al*, 2017; Shin & Brangwynne, 2017; Ditlev *et al*, 2018; Lyon *et al*, 2021). With linear dimensions in the biological mesoscale (between ~100 nm and a few micrometers), compartments are supra‐molecular, i.e., larger than their individual macromolecular components. Typically, tens or even hundreds of different macromolecular species, usually in multiple copies, populate each individual compartment and interact in it, conferring different degrees of internal order on compartments (Wu & Fuxreiter, 2016; Goetz & Mahamid, 2020; Peran & Mittag, 2020; Fare *et al*, 2021; Korkmazhan *et al*, 2021).

**Figure 1:**
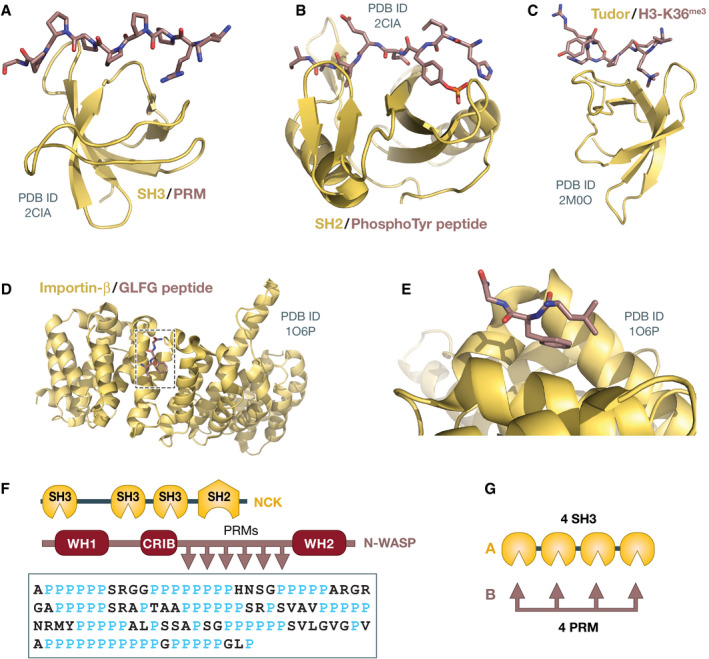
Examples of site‐specific interactions and their combination in MVPs (A–E) Cartoon diagrams of various protein domains and proteins discussed in the text (yellow), with their cognate ligands shown in stick. (A) Src homology 3 (SH3) domain with proline‐rich motif (PRM); (B) Src homology 2 domain with tyrosine‐phosphorylated peptide; (C) Tudor domain with peptide from histone H3 trimethylated on lysine 36; (D) Importin‐β with Gly‐Leu‐Gly‐Phe (GLGF) peptide; (E) Enlargement of area boxed in D. The protein data bank (PDB) codes are indicated; (F) Domain organization of the NCK and N‐WASP proteins, and sequence of the proline‐rich region of N‐WASP (Uniprot, human sequence); and (G) Artificial multivalent constructs used by Li *et al* (2012).

The physicochemical drivers of the biogenesis, maintenance, and disassembly of compartments, and their ability to concentrate macromolecules without an encapsulating membrane, have attracted considerable interest (Banani *et al*, 2017; Shin & Brangwynne, 2017). Molecular mechanistic biology, with its focus on the identification, characterization, and visualization of discrete and highly specific interactions of macromolecules, has uncovered how the building blocks of compartments assemble and interact reciprocally (see Fig 1A–E for a few examples) (Jones & Thornton, 1996; Sudha *et al*, 2014). How the building blocks interact to give rise to mesoscale structures devoid of strong internal order, however, is less well understood and harder to model.

**Figure 2:**
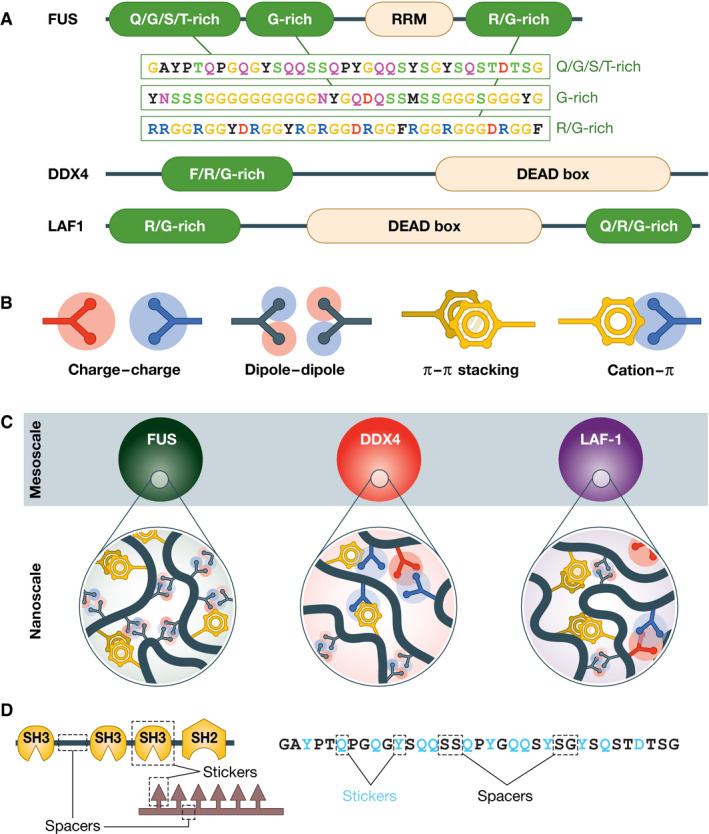
Examples of IDPs and low‐affinity, non‐site‐specific interactions (A) Domain organization of three IDPs and specific sequence stretches from each of three low‐complexity regions of human FUS; (B) Examples of low‐affinity interactions believed to drive homotypic PS of IDPs; (C) Droplets of FUS, DDX4, and LAF‐1 (indicated as “mesoscale”) are proposed to arise from multiple nanoscale interactions shown in B; (D) Sticker‐and‐spacer model for interactions of MVPs (left) and of IDPs (right). Gln and Tyr (Q and Y, respectively) are considered stickers that could interact through dipoles and stacking. The entire figure is an adaptation of Fig 2 from Brangwynne *et al* (2015).

In recent years, a burgeoning new field of research in cell biology, biomolecular phase separation (PS), has promoted a radically different explanation for how membraneless compartments assemble. Under the PS paradigm, classical specific macromolecular interactions are attributed a secondary role in compartment assembly. Compartments are rather proposed to form under the action of one or a few specific PS drivers (almost invariably proteins). Drawing concepts from polymer chemistry (Hyman *et al*, 2014; Brangwynne *et al*, 2015), PS drivers are treated as *associative polymers* that phase‐separate thanks to transient, low‐affinity, cohesive interactions (Fig 2B and C) (Tanaka, 2011; Hyman *et al*, 2014; Brangwynne *et al*, 2015; Uversky *et al*, 2015; Banani *et al*, 2017; Shin & Brangwynne, 2017; Boeynaems *et al*, 2018; Choi *et al*, 2020).

The PS paradigm proposes that membraneless compartments are (usually) liquid like and that they arise from liquid–liquid (LL) demixing, a process exemplified by the spontaneous, thermodynamically driven separation of oil from water caused by poor solvation (Hyman *et al*, 2014; Banani *et al*, 2017). This idea was ignited by provocative observations that P‐granules and nucleoli form droplets that fuse, wet neighboring cellular structures, drip, and display rapid internal mobility of its components when assayed by fluorescence recovery after photobleaching (FRAP) (Phair & Misteli, 2000; Brangwynne *et al*, 2009, 2011). Subsequent studies on two classes of molecules frequently identified in membraneless compartments, multivalent proteins (MVPs; Fig 1F and G) and intrinsically disordered proteins (IDPs), containing low‐complexity domains (LCDs, examples of which are shown in Fig 2A), began to provide a molecular and theoretical underpinning to the PS paradigm. For instance, natural or engineered MVPs were shown to form droplets *in vitro* when highly concentrated (Li *et al*, 2012; Banani *et al*, 2016; Harmon *et al*, 2017), and similar observations were also made with various IDPs [e.g., (Kato *et al*, 2012; Elbaum‐Garfinkle *et al*, 2015; Lin *et al*, 2015; Molliex *et al*, 2015; Nott *et al*, 2015; Pak *et al*, 2016)]. These observations supported the progressive identification of MVPs and IDPs as *scaffolds* driving PS through *weak homotypic interactions* (i.e., involving the same macromolecule), while other macromolecules attracted to the same compartments through *heterotypic interactions* (i.e., involving different types of macromolecules) were identified as *clients* (Hyman & Simons, 2012; Banani *et al*, 2016, 2017). Collectively, these developments caused the transformation of PS from the abstractions of a useful analogy to a seemingly coherent and unifying paradigm for the biogenesis of membraneless compartments from the nano‐ to the mesoscale (Hyman & Brangwynne, 2011; Banani *et al*, 2017).

## Macromolecular interactions

Below, we will often refer to three main types of macromolecular interactions. The first (type I) are homotypic or heterotypic interactions established (mainly) by IDPs, i.e., by proteins, or segments thereof, that do not adopt a stable folded conformation. IDPs are usually viewed as arrays of stickers and spacers, with spacers determining overall solubility, and stickers mediating interactions (Tompa & Fuxreiter, 2008; Brangwynne *et al*, 2015; Martin & Mittag, 2018; Choi *et al*, 2020; Borcherds *et al*, 2021; Fare *et al*, 2021). Stickers are capable of only few, relatively low‐affinity and poorly specific types of attractive interactions, including charge–charge, dipole–dipole, cation Π, and Π–Π stacking (Fig 2B).

The second and third types of macromolecular interactions (types II and III) are also homotypic or heterotypic, but are enabled by the specific three‐dimensional arrangement (reflecting conformation) and the detailed chemical identity of the binding interfaces. These interactions involve at least one folded domain of a macromolecule and either (type II) a short linear segment of a target macromolecule, which may also fold locally in the process of binding, or (type III) another folded domain. While type I interactions are mediated by a limited set of attractive or repulsive bonds, with the result that their specificity is limited, macromolecular interactions of types II and III usually involve large, conserved surface patches on the interacting macromolecules that exploit the spatial and chemical complementarity of the binding interfaces as well as steric exclusion (Jones & Thornton, 1996; Aloy *et al*, 2003; Choi *et al*, 2009; van der Lee *et al*, 2014).

The PS idea identifies interactions of type I typically as *fuzzy*, whereas interactions of types II and III are now often defined *stereospecific* (Hyman & Brangwynne, 2011; Banani *et al*, 2017). The term *stereospecific* is used in chemistry to denote formation, recognition, or reaction of enantiomers. If used with reference to biological interactions, the term *stereospecific* aims to put emphasis on the property that specificity is largely dictated by the complementary geometry and chemistry of binding sites. This use of *stereospecific* is therefore related to, but deviates considerably from, the traditional definition of the term. For this reason, here I prefer using simply *site‐specific interaction* (abbreviated as SSI) to refer to interactions of types II and III.

SSIs are enormously versatile, as masterly described in a classic review (Mammen *et al*, 1998). As a rule of thumb, they are titrated between 0.1 and 10 times the dissociation constant (K_D_) (Jarmoskaite *et al*, 2020). If the K_D_ is sufficiently small (high affinity), correspondingly low concentrations of binders, normally well below their solubility limit, will suffice. SSIs can be easily and rapidly modulated, e.g., through post‐translational modifications, without having to change the concentration of binders. K_D_s can be extremely low, to the point of making an interaction essentially irreversible, as in the core of many multisubunit macromolecular complexes, or relatively high for regulated interactions, and easily adjustable to non‐equilibrium conditions in active processes. Thus, while in principle, both type I and type II/III interactions can reach high affinity (e.g., for type I interactions by harnessing multivalency), the hallmark that distinguishes I from II and III is the low specificity of the first and the high specificity of the latter two.

## Condensates

As discussed below, the narrative that compartments are liquid, and driven by low‐affinity, low‐specificity fuzzy interactions has severe shortcomings. Nonetheless, it has exercised a tremendous influence on the field’s developments. Briefly, after disregarding SSIs as potential drivers of compartment biogenesis on the basis of a supposed incompatibility with the liquid‐like appearance of phase‐separated compartments (Hyman & Brangwynne, 2011), it licensed PS assays in which putative PS drivers were studied in perfect isolation *in vitro*, and usually confirmed in this presumed role (prematurely, as explained below). This allowed PS, initially invoked for the assembly of nuclear and cytoplasmic bodies involved in ribonucleoprotein (RNP) assembly and metabolism, such as nucleoli and P‐granules, to claim for itself an ever‐growing list of cellular territories. PS is now considered a self‐evident universal driver of compartment assembly, as implied by the proposal to rename all membraneless compartments *biomolecular condensates* (Banani *et al*, 2017; Shin & Brangwynne, 2017; Snead & Gladfelter, 2019) on the basis of their *ability to concentrate molecules*, that *they comprise biological macromolecules*, and that they may arise *through similar mechanisms* [text in *italic* indicates textual citations (Banani *et al*, 2017)]. This definition encompasses functionally and compositionally diverse compartments like centrosomes, centromeres, kinetochores, transcriptional super‐enhancers, chromatin domains, chromosomes, sites of response to DNA damage and DNA recombination, membrane‐associated signaling complexes (which of course are not “membraneless” *per se*, but nevertheless not surrounded by a membrane), and many more (Hyman & Brangwynne, 2011; Banani *et al*, 2017; Shin & Brangwynne, 2017; Lyon *et al*, 2021).

Not unlike the term *stereospecific*, also the term *condensate*, as often used in the PS context, carries some ambiguities. Condensation is usually taken to describe the transition of a gas to a liquid, or chemical bond formation between two molecules accompanied by release of water. In PS publications, the term *condensation* is rather used to imply concentration of biomolecules through a phase change (Banani *et al*, 2017)—which may be considered a similar analogy as using *condensation* to describe the compaction of chromosomes upon mitotic entry. It is important to keep in mind, however, that the ability to concentrate molecules, e.g., to accelerate reactions or inhibit them through sequestration (Banani *et al*, 2017; Shin & Brangwynne, 2017; Lyon *et al*, 2021), is an attribute that does not support a role of PS more than it supports other mechanisms of biogenesis. SSIs can do that too, and better, as I will discuss. Furthermore, while concentration of specific macromolecules is clearly a feature of membraneless compartment, whether cellular compartments generally reach overall macromolecular concentrations higher than those of the surrounding cellular medium is unclear (Handwerger *et al*, 2005; Wei *et al*, 2017). Thus, collectively, *condensate* should not be taken as a literal descriptor of mesoscale membraneless compartments, at least in relation to our current knowledge of the processes that promote their assembly.

## What drives compartment assembly?

Another question raised by the definition of *biomolecular condensates* is whether it is true that the *similar mechanisms* from which compartments are proposed to arise imply homotypic fuzzy interactions of PS scaffolds. These interactions are assumed to be the basis of PS and compartment assembly, but as we shall see, this is rather implausible and defies compelling evidence that the only truly general mechanism of compartment biogenesis involves highly specific *heterotypic* SSIs occurring at concentrations well below the saturating concentration (*C*_sat_) of any of the interacting components, i.e., in the one‐phase regime. These concerns, to the extent that they call into question the very arguments on which the PS concept has built its success, are highly significant and require thorough motivation. In this essay, I will therefore examine two crucial questions: (i) Are low‐specificity, fuzzy interactions of macromolecules as associative polymers literally the primary physicochemical driver of biogenesis of membraneless compartments in cells under physiological conditions (I will refer to this as *general PS*)? and (ii) Can phase transitions influence a compartment’s solvation and material properties *after* macromolecules have become concentrated there by more traditional binding mechanisms (I will refer to this as *special or restricted PS*)? Below I will first discuss arguments indicating that the answer to question 1 is *(most) likely no*. Later, I will also discuss why the answer to question 2 is instead *likely yes*, but probably for a relatively small subset of compartments. I will also clarify why demonstrating special PS, l*et al*one general PS (if it exists in our cells), requires standards of proof that studies on PS have almost invariably ignored.

## What is general PS?

General PS assumes that low‐specificity homotypic interactions of one or very few PS scaffold macromolecules drive, at concentrations *above* their *C*_sat_, a *density transition* that results in the formation of coexisting dilute and dense phases, each with a fixed concentration of the scaffold (Banani *et al*, 2017). The dense phase may further promote concentration of other species to complete assembly of a mature compartment (Hyman *et al*, 2014; Banani *et al*, 2017). Examples adapted from Brangwynne *et al* (2015) are shown in Fig 2C.

As noted, MVPs and IDPs were initially identified as likely scaffolds in general PS. Their plausibility as PS drivers reflected (i) their enrichment in various membraneless compartments and (ii) their tendency, especially for the IDP class, to be aggregation prone and implicated in various degenerative pathologies (Alberti & Hyman, 2021). This ignited an extensive program of study of their phase behavior *in vitro*, with attempts to correlate it with their behavior in living cells and in disease. For instance, studies focused on the associative properties of tight complexes of reciprocally interacting linear MVPs, including engineered ones containing multiple SH3 domains and proline‐rich motifs (PRMs), separated by flexible linker spacers (Fig 1F and G) (Li *et al*, 2012; Banani *et al*, 2016; Harmon *et al*, 2017; Case *et al*, 2019).

While IDPs may be viewed as *assemblers*, whose distributed linear motifs engage in SSIs with target folded domains (type II interactions) (van der Lee *et al*, 2014), the PS paradigm considers IDPs usually as associative polymers with alternating stickers and spacers (type I) (Choi *et al*, 2020). The same sticker‐spacer description may be applied to MVPs, where folded domains are stickers‐mediating interactions and the linkers between them are spacers (Choi *et al*, 2020). This description aims to underline a unifying similarity of MVPs and IDPs that might explain why both classes drive PS (Fig 2D). However, the similarity is less obvious than implied by this unifying description, as discussed more thoroughly below, because the stickers in MVPs usually are SSIs that can generate very significant binding affinities and specificity, whereas the stickers of IDPs are relatively low affinity and non‐specific, at least in non‐aggregative states (Wu & Fuxreiter, 2016; Korkmazhan *et al*, 2021).

## The unintuitive general PS

**Figure 3:**
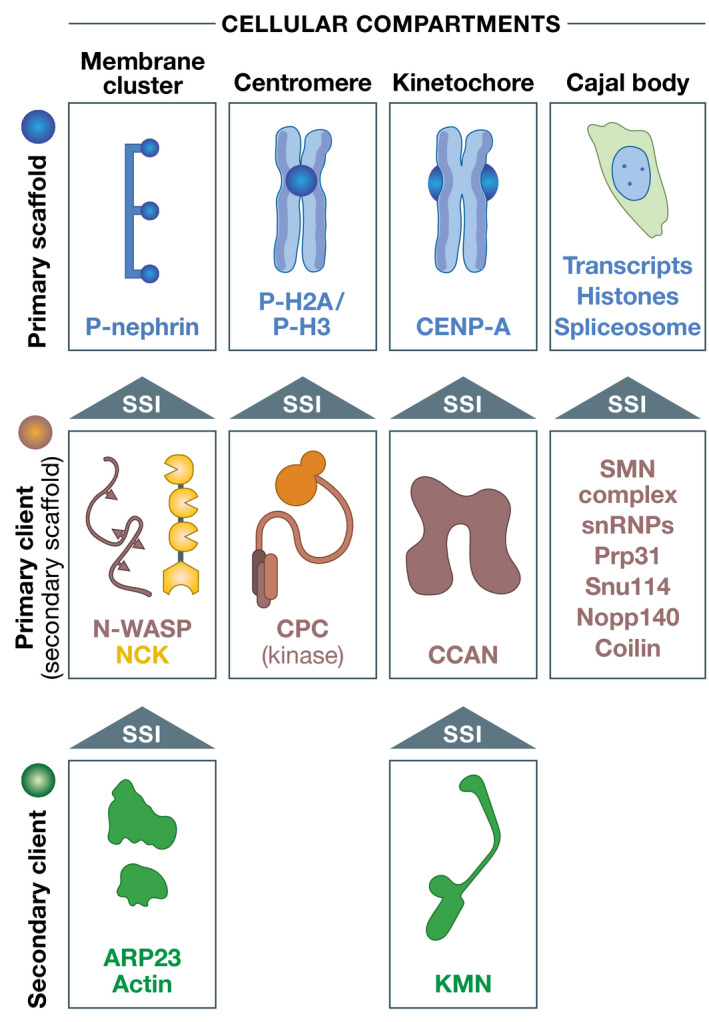
Hierarchy of compartment assembly Compartments are usually hierarchical. Nephrin, as an element of membrane clusters, is a transmembrane protein whose intracellular domain undergoes regulated phosphorylation. As primary scaffold, it acts as a binding site for the NCK client through its SH2 domain. N‐WASP binds NCK through phosphorylation. Regulators of actin polymerization may be further recruited to clusters. During mitosis, the CPC kinase complex is recruited to specific phosphorylated residues of histone H2A and H3 that are enriched in the centromere region. It is therefore a client of the centromere, although it has been suggested to act as scaffold in PS. The kinetochore assembles on the histone H3 variant CENP‐A, which is part of a specialized centromeric histone complex. Constitutive centromere‐associated network (CCAN) subunits are recruited through interactions with CENP‐A. The Knl1‐Mis12‐Ndc80 (KMN) complex is recruited to CCAN through established SSIs. Not shown are tertiary clients like the spindle checkpoint proteins BUB1 and MAD2 discussed in the text. In Cajal bodies, specific RNAs transcripts, for instance, of histones, but also spliceosome subunits, act as scaffolds for the recruitment of a variety of downstream proteins (Kaiser *et al*, 2008; Shevtsov & Dundr, 2011). The recruitment hierarchy remains poorly understood. Other nuclear bodies, like the histone body or the nucleolus, also require transcription (see main text for details).

Fascinating as they may seem, the PS tenets prove on closer scrutiny less intuitive than the simple oil–vinegar analogy suggests. Proteomes may have evolved to maximize specificity and minimize aggregation of proteins (Zhang *et al*, 2008; Johnson & Hummer, 2011). Neither specificity nor aggregation seem to be of significant concern for proposed general PS mechanisms of condensate biogenesis, which rather focus on low‐affinity and low‐specificity interactions (Fig 2) at or above the solubility limit of one or more PS drivers (Hyman *et al*, 2014; Brangwynne *et al*, 2015). It is hard to imagine how fuzzy interactions of very low specificity would promote selective, thermodynamically driven condensation of any particular macromolecule at typical cellular concentrations, with myriad competing interactions of a similar kind. And how can the same limited set of weak, non‐site‐specific interactions explain how different compartments maintain their identities and prohibit or enable co‐PS? Are active processes involved in this complicated scheme, and if so, how? How is the saturating concentration of every putative PS driver adjusted across different specialized cells with different sizes and compositions of their cytosol, and even more so across different organisms? And how would the formation of toxic aggregates be controlled given the proposed generality of this mechanism for compartment biogenesis? Let us also consider that compartments usually form around defined spatial and temporal cues, delimited by the presence of a *primary* scaffold (Fig 3), and ideally do not extend beyond them. These cues, be it signaling‐active transmembrane receptors or centromeres, attract and concentrate the correct targets almost certainly through SSIs with adequate affinity and specificity, probably also dictating the final concentration of various compartment’s components. If PS drivers were indeed sufficient for PS in the absence of spatial cues, why do they not assemble unrestrained in space and time?

## Liquid and site specific

Thus, general PS may seem to counter biological intuition when it proposes an inverted hierarchy of compartment assembly that prioritizes unspecific interactions over more probable site‐specific ones (Hyman & Brangwynne, 2011). If in all evidence macromolecules usually become concentrated in their target cellular locales due to highly site‐specific interactions, why were these not considered as a mechanism of biogenesis? The primary answer is that they were perceived as incompatible with the liquid‐like behavior of supposed phase‐separated organelles (Hyman & Brangwynne, 2011). For instance, the kinetics of recovery of many components of compartments in FRAP experiments was deemed too fast for SSIs (Hyman & Brangwynne, 2011). This conclusion appears premature. The kinetics of typical SSIs, as they may be captured by half‐time of the bound state at equilibrium, t_1/2_, are well within boundaries demonstrated by FRAP experiments on liquid‐like compartments [for a more thorough and informative discussion on the execution and interpretation of FRAP experiments, including more sophisticated protocols to assess the potential presence of PS, please consult Erdel *et al* (2020; McSwiggen *et al* (2019b); Sprague and McNally (2005); Taylor *et al* (2019) and references therein]. If a macromolecule bound to a receptor in a compartment (rather than getting there through condensation), its FRAP recovery rates would be typically determined by the dissociation rate constant (*k*_off_), i.e., by the rate of release of the bleached molecules from their receptor, necessary for replacement with new fluorescent molecules (Sprague & McNally, 2005). The faster the bleached molecules dissociate from their receptor, the faster the recovery.

**Figure 4:**
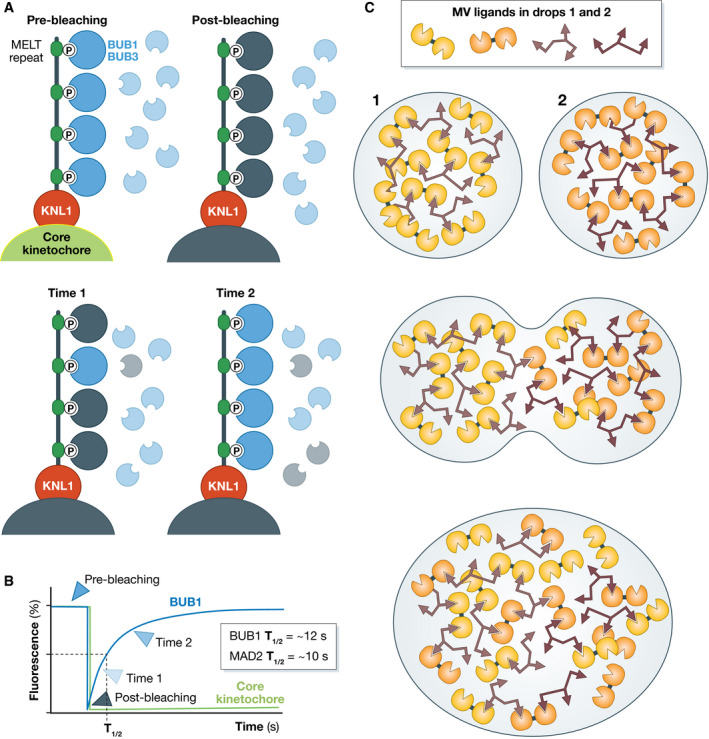
Liquid like does not exclude SSIs (A) KNL1, an IDP at kinetochores, contains multiple sequence‐related phosphorylation sites for the recruitment of the BUB1/BUB3 complex, where BUB3 is a phospho–amino acid adaptor. KNL1 docks to CCAN in the core kinetochore. A FRAP experiment on BUB1/BUB3 and on a core kinetochore subunit would lead to fundamentally different conclusions on the nature of the compartment, as the core subunit do not exchange and would not recover, whereas BUB1/BUB3 would exchange in seconds; (B) Hand‐drawn curves representing the recovery behavior shown in A; (C) Two imaginary directly interacting MVPs in neighboring phase‐separated droplets (1 and 2, where the small differences in color are meant to recognize the origin of molecules in the original droplets) could easily mix if the interaction times of the individual modules allowed relatively rapid exchange.

For example, the spindle assembly checkpoint (SAC) complex BUB1/BUB3 binds its kinetochore receptor with a dissociation constant (K_D_) of approximately 100 nM and a t_1/2_ of recovery in FRAP experiments of ~10 s (Overlack *et al*, 2015, 2017) (Fig 4A and B). Similarly, FRAP recovery rates and fractions for another SAC protein at kinetochores, MAD2, were initially measured in living cells and then reproduced with recombinant proteins in a reconstituted system *in vitro*, with typical t_1/2_ of recovery of a few seconds (Howell *et al*, 2004; Shah *et al*, 2004; Vink *et al*, 2006). Other kinetochore components (indicated as “core kinetochore” in Fig 4A) remain connected to the underlying chromatin throughout a cell’s life, without ever exchanging (Jansen *et al*, 2007). At kinetochores, both rapid and slow/non‐existent turnovers reflect SSIs (Fig 4) (Musacchio & Desai, 2017).

In FRAP experiments, predicted recovery half‐times of macromolecular interactions, just assuming typical K_D_s for reversible interactions and typical association rate constants (Shammas *et al*, 2012), will thus extend from sub‐seconds to hours, considered to indicate a diffusive liquid‐like state or even solid‐like state in many claims of PS [typically 1–100 s (McSwiggen *et al*, 2019b)]. If SSIs turn over rapidly, as they can, they provide a plausible basis for another property of liquid‐like membraneless compartment, their fusion. Turning over rapidly, new “mixed” interactions in fusing droplets will progressively replace the identical interactions that existed in the individual droplets, especially if the network is heavily cross‐linked through interactions turning over rapidly, as shown in a hypothetical example in Fig 4C [see also discussion in (Erdel & Rippe, 2018)]. In cells, active processes may additionally contribute to promote fusions, as observed for mitotic spindles, large ensembles of microtubules, microtubule cross‐linkers, and molecular motors engaged in many reciprocal dynamic SSIs, which can fuse within a few minutes (Gatlin *et al*, 2009).

Finally, how surface tension/energy of membraneless compartments emerges, and which compartments should or should not show surface tension and sphericity, regardless of the detailed mechanism of biogenesis, are open question on which agnosticism is due [see Erdel and Rippe (2018); McSwiggen *et al* (2019a); McSwiggen *et al* (2019b); Peng and Weber, (2019) for further discussions]. It may be speculated that surface energy emerges from the density of the underlying mesh of interactions. Furthermore, we may surmise that macromolecules at the surface of a putative condensate have higher energy than those inside it. In this case, the surface energy may be expected to be high—and the boundary sharp—when the binding energies of the underlying molecular interactions are strong, which usually implies site specificity.

## Most compartments may not result from PS

Thus, there are no good reasons to exclude SSIs as drivers of compartment assembly. Moreover, they are significantly more likely to participate in the biogenesis of the great diversity of subcellular compartments than a restricted variety of low‐affinity unspecific homotypic interactions. By way of example, the kinetochore is a compartment on chromosomes that concentrate at least 60 distinct polypeptides to control chromosome segregation (Musacchio & Desai, 2017). With the dissection of kinetochores well underway, all interactions characterized so far appear to be site specific, with discrete binding interfaces, whose mutation predictably prevents recruitment of downstream components. This does not reflect an absence of MVPs or IDPs, as several kinetochore components, including CENP‐C^Mif2^ and KNL1^Spc105^, belong to these classes. In addition, we have no evidence that the kinetochore acts as an unspecific “sink” for functionally unrelated macromolecules. Its composition seems to be limited to functional components recruited to it in a site‐specific manner, as shown for BUB1/BUB3 and MAD2. This may be the norm, as the compositional identity of compartments is usually well defined. Readers are referred to a discussion on the centrosome as a condensate (Woodruff *et al*, 2017; Raff, 2019), and contextually to a report on how artificial coacervation promotes filamentation of a bacterial tubulin homolog (Te Brinke *et al*, 2018).

Seen retrospectively, it may seem puzzling that SSIs were excluded as an underlying possible driver of compartment assembly. Why were they? I suspect the answer is that the field, since early on and progressively more pervasively, accepted that compartments concentrate macromolecules through a mechanism of PS, and continued to support this idea, often in presence of very strong evidence to the contrary, without a rigorous test of the hypothesis, as I will show below. My concerns extend and complement previously formulated objections that many compartments indicated as *condensates* lack features expected if PS were their driver (Wheeler *et al*, 2016; McSwiggen *et al*, 2019b; Erdel *et al*, 2020). If many compartments do not show hallmarks of PS, and if their biogenesis is likely to reflect mechanisms other than those advocated by its proponents, we should avoid calling them condensates, which should be rather reserved for compartments with evident signs of condensation by PS.

## Validation pipeline: part 1

**Figure 5:**
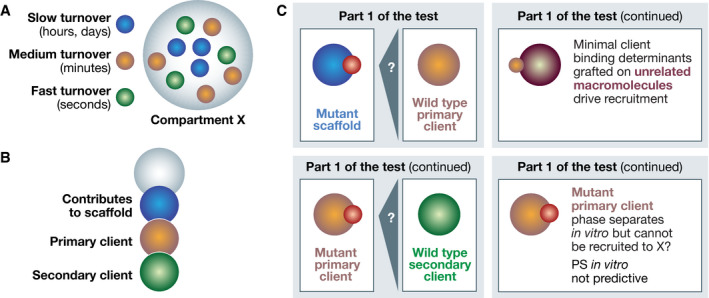
Interactions in an imaginary compartment (A) Compartment X concentrates several components, three of which are shown in blue, brown, and green colors together with their turnover times; (B) An assembly hierarchy might have been identified while investigating the interactions in Compartment X; (C) *Upper left*: Can a primary client with wild‐type sequence be recruited to a scaffold with mutations in a binding site for the primary client? If so, the binding interaction is at least necessary for the recruitment. *Upper right*: Grafting of binding site of primary client for scaffold allows recruitment of unrelated macromolecule. Minimal binding sites indicate sufficiency. *Bottom left*: Iterative analysis down the interaction hierarchy. *Bottom right*: A mutant client that fails to be recruited to X but undergoes PS *in vitro* like the wild‐type counterpart indicates PS *in vitro* not predictive.

So, there are compartments whose predominant and possibly only drivers of assembly are SSIs. Are there any compartments where PS is an undisputable driver of assembly? How should we investigate this question for other compartments where our knowledge of the assembly mechanisms is limited? As a thought experiment, let us consider a newly discovered membraneless compartment X with several concentrated macromolecules (Fig 5A). Measured FRAP rates and recovery fractions range from essentially no recovery for certain components to full recovery in minutes or seconds for others. These FRAP rates may be taken as an indication that condensate X is partly solid, partly liquid, but also as an indication that SSIs of variable strength are at stake. With hypotheses facing off, what should be done?

Using a combination of *in vitro* and *in vivo* work, SSIs supporters should aim to identify all components of compartment X and their binding regions, mutate them, and show which are crucial for assembly and what hierarchy exists, if any (Fig 5B). What should PS supporters do? Their hypothesis is that solvation exemplified by oil–water is crucial for compartment assembly, *irrespective of any existing site‐specific interaction with other components*. As it first needs to be proven that SSIs are at least *insufficient* for compartment assembly, one needs to identify, as a premise, all possible SSIs to assess how they contribute to assembly. This is precisely what those supposing SSIs planned to do, and that is how it should be: all possible hypotheses need to be considered, and SSIs should not be excluded from consideration without compelling reasons.

With the characterization of compartment X in progress, SSIs might have or have not been found. If they had not been found, compartment X might qualify for general PS. If they had been found, there would be a prospect for special PS but also for no PS at all. Thus, if SSIs have been found, the next question is whether they are necessary (and ideally sufficient) for compartment assembly *in vivo*. To assess this, a putative assembly scaffold may be mutated to eliminate the site‐specific binding determinant for a client (Fig 5C). In a strategy enabled by separation‐of‐function mutants, the mutant scaffold will assemble in its correct location if there remain binding sites for the compartment. If the client fails to become recruited, the SSI is at least necessary for recruitment in compartment X. This could be reapplied to the chain of primary, secondary, and higher‐order scaffold/clients. Under the PS paradigm, the continued recruitment of the mutant primary scaffold would imply that its solubility has not changed appreciably. If recruitment of the client, on the other hand, is abrogated, the PS model cannot invoke changes in its solubility in the bulk phase, as its sequence is invariant (the mutation is on the scaffold). Conversely, one may also mutate a client to prevent its recruitment into X, and ask if the mutation affects its PS behavior *in vitro*, i.e., whether PS *in vitro* is predictive of localization. In addition, as a criterion for sufficiency of SSIs, one may isolate the binding determinants of a client and graft them onto a macromolecule with entirely different features to ask if it becomes recruited in X, which would imply that the compartment does not discriminate for or against other features of the client (such as those that may determine its solubility).

Collectively, the pipeline described above is Part 1 of the test. With compartments being usually very complex, identifying interactions and assessing assembly hierarchies is an extremely demanding task requiring great biochemical prowess and a lot of patience, yielding modest short‐term rewards; but nonetheless essential to real progress.

## Validation pipeline: part 2

With Part 1 complete, PS may be ready for limelight if SSIs in X were found to be completely absent, or if they were unnecessary or insufficient toward its biogenesis (in order of increasing likelihood). Part 2 of the test should aim to gather hard evidence for PS. The general PS idea implies that a condensate will appear when the concentration of a putative PS driver exceeds *C*_sat_ (implying there should be a recognizable *C*_sat_). To test PS, we cannot manipulate the SSIs we might have identified in Part 1 of the test, as they are crucial thermodynamic parameters that have to remain unaltered when we are trying to assess *additional effects* of PS. Thus, we can only aim to change *C*_sat_, for instance, by increasing the relative concentration of candidate components of X in the dilute bulk phase so that they would not join X, either because they prefer it less or because they prefer the dilute phase more. But *how* is *C*_sat_ determined in a cell? We have powerful theories for the behavior of associative polymers in “poor solvent” conditions (Hyman *et al*, 2014; Brangwynne *et al*, 2015; Harmon *et al*, 2017). But what about the cytosol, enormously complex, crowded, and offering a variety of unpredictable opportunities for competitive short‐lived unspecific interactions (Rivas & Minton, 2018)? The dense phase is also a complex compartment offering a variety of opportunities for short‐lived unspecific interactions. What determines the solubility in the dilute and condensed phases for any individual component? Is there a hard criterion for defining sufficiency? What exactly phase‐separates and defines the putative condensate? A single macromolecule? Its network of interactors? All components, as it seems plausible? How is a scaffold–client relationship maintained in this framework? How do post‐translational modifications influence this process? And are there additional energy‐consuming processes that further complicate the picture?

It is evident that if these questions were to be answered, Part 2 of the test would soon become an extremely complex endeavor, enormously more complex than the homotypic interaction assays that have been relied upon to prove general PS. In the majority of studies where general PS was claimed, SSIs were never considered as potential drivers of compartment biogenesis, even if clear evidence of their involvement already existed (two among many other cases are briefly discussed below). With Part 1 of the test generally skipped altogether, a poorly predictive and oversimplified version of Part 2 of the test made it all but impossible to fail. How is Part 2 of the test run in practice? Typically, it begins with the nomination of a plausible candidate for an associative polymer: MVPs, IDPs, or their fragments, but more recently essentially any macromolecular class. With experiments that sometimes demonstrate impressive technical and analytical depth, the putative PS driver is subjected to experiments *in vitro* aimed at deciphering its *phase grammar* and the rheological properties of the resulting droplets [e.g., Molliex *et al* (2015); Nott *et al* (2015); Pak *et al* (2016); Patel *et al* (2015); Wang *et al* (2018)]. If PS is observed and mutations that perturb it can be identified, which is almost invariably the case, the protein or its fragment is usually overexpressed in a cell together with mutants to build evidence that cellular behavior matches the results *in vitro*. In a majority of cases, the questions of *how* solubility of a putative PS scaffold is determined *in vivo* and whether a constant saturation concentration can be measured are not considered (see below).

## Are general PS tests predictive?

This validation procedure, which has already led to the proposition of a large number of supposed PS “scaffolds,” has profound shortcomings (Protter *et al*, 2018). First, at sufficiently high concentrations, and often with help of crowding agents, there are virtually infinite conditions *in vitro* with the potential to modulate the solubility of a macromolecule until it crystallizes (rarely), or it precipitates or phase separates in droplets (less coveted, at least by crystallographers, but frequent) (Dumetz *et al*, 2008). These experiments can hardly be considered predictive of cellular behavior.

Second, it is unclear if the species that phase‐separates *in vitro* is a sufficiently faithful representation of the cellular counterpart. For example, divergence may result from incomplete modifications, proteolysis, conformational variability, or because the protein *in vitro* binds to a prominent contaminant, such as a chaperone. Verifying the precise chemical composition, conformation, and degree of homogeneity of a purported phase‐separated macromolecule in a cell is essentially unfeasible with our current means. Is the species in the compartment truly identical to its diluted counterpart? Is it homogenous? Is it precisely the same as it was tested *in vitro*?

Third, we should be wary of the way the scaffold–client conceptualization is molded to build a criterion for PS sufficiency. Many macromolecules identified as PS scaffolds are considered clients in the binding paradigm. For instance, NCK and N‐WASP are binding clients of phosphorylated nephrin (see below). Should these agents be sufficient for PS in cells as implied by their PS *in vitro*, they would assemble signaling compartments without activation cues, likely with catastrophic consequences.

Fourth, phase‐separated droplets *in vitro* may seem to mimic compartments observed in cells, but they only represent an initial, pre‐equilibrium stage of a process (Ostwald ripening, caused by minimization of the surface energy) that on a longer timescale promotes formation of a single macroscopic dense domain, an unappealing representation for most cellular compartments.

Fifth, how to interpret the outcome of PS experiments *in vitro* is uncertain and contentious. What significance should be attributed to (i) the formation of dense hydrogels, (ii) liquid droplets, (iii) cross‐β sheet interactions preluding to amyloid aggregation, or (iv) the various time‐dependent drop‐hardening phenomena [see, for instance, (Ader *et al*, 2010; Han *et al*, 2012; Kato *et al*, 2012, 2021; Burke *et al*, 2015; Kroschwald *et al*, 2015; Molliex *et al*, 2015; Nott *et al*, 2015; Patel *et al*, 2015; Xiang *et al*, 2015; Brady *et al*, 2017; Kato & McKnight, 2018; Martin & Mittag, 2018; Wang *et al*, 2018; Murthy *et al*, 2019; Martin *et al*, 2020; Borcherds *et al*, 2021)], for the actual structure and function of a condensate *in vivo*?

Finally, even if we had observed some correlation between the phase behavior of certain constructs *in vitro* and *in vivo*, we would remain entirely ignorant of how solubility of our putative driver is determined in the cell. There really is no good reason to expect that the solubility of putative PS drivers cherry‐picked for *in vitro* experiments matches their solubility in the cell. Additionally, the passive, thermodynamically driven experiment *in vitro* needs to confront the possibility of energy‐consuming, active processes present in (probably most) compartments, and possibly of stress responses caused by overexpression of constructs in the *in vivo* experiments.

## Acceptance of complexity

Thus, the PS sufficiency tests that swaths of papers have employed to identify PS drivers greatly oversimplify the description of the mechanism of compartment assembly, and ought to have been given more credit for what they *might imply* than for what they effectively say. The infallibility of PS imputations based on these tests are a warning that validation standards lack robustness, but until recently these assays have been given full credit in major reviews (Brangwynne *et al*, 2015; Alberti *et al*, 2019). That these experiments are poorly predictive is exemplified by the complex effects of RNAs and other binding partners on PS of putative drivers, including FUS, TDP43, G3BP1/2, and many others [e.g., (Han *et al*, 2012; Schwartz *et al*, 2013; Burke *et al*, 2015; Kroschwald *et al*, 2015; Molliex *et al*, 2015; Patel *et al*, 2015; Zhang *et al*, 2015; Feric *et al*, 2016; Langdon *et al*, 2018; Maharana *et al*, 2018; Protter *et al*, 2018; Wang *et al*, 2018; Mann *et al*, 2019; Guillen‐Boixet *et al*, 2020; Riback *et al*, 2020; Sanders *et al*, 2020; Yang *et al*, 2020; Roden & Gladfelter, 2021)]. Only limitedly to FUS, for instance, its purported PS is inhibited by ATP, cell lysates, and RNA (Patel *et al*, 2017; Maharana *et al*, 2018; Protter *et al*, 2018). A possible suggestion to overcome the limits of the greatly error‐prone *in vitro* solubility tests for general PS currently in use is to run solubility experiments in *cytomimetic media* (Protter *et al*, 2018; Rivas & Minton, 2018, 2019; Gnutt *et al*, 2019; Nakashima *et al*, 2019). These should be realistically concentrated (100 mg/ml and more) and devoid of components of the putative condensate (else PS sufficiency cannot be demonstrated), *e.g*., bacterial cytosols or a realistic artificial medium built from the most dominant components of eukaryotic cytosols at their typical overall concentrations (Beck *et al*, 2011).

## The multiphase dilution of PS

Recent work on the biogenesis of nuclear bodies, including the Cajal body and the nucleolus, and of other RNP compartments demonstrates that initial interpretations on the role of PS in their biogenesis were premature. The assembly of nuclear bodies mobilizes SSIs between various protein components and specific *cis*‐acting sequences on specific RNAs, and requires active transcription (Savino *et al*, 2001; Leung *et al*, 2004; Shav‐Tal *et al*, 2005; Kaiser *et al*, 2008; Shevtsov & Dundr, 2011; Grob *et al*, 2014; Berry *et al*, 2015; Caudron‐Herger *et al*, 2016; Falahati *et al*, 2016; Jain *et al*, 2016). Conversely, there does not seem to be strong biological evidence that these bodies originate from general PS. Nonetheless, this view has become dominant since it was shown that putative scaffolds in the nucleolus, nucleophosmin/NPM1, and fibrillarin, preferably in presence of RNA, phase‐separate *in vitro* to scaffold the assembly of immiscible phases reminiscent of the granular and dense fibrillar components, respectively (Brangwynne *et al*, 2011; Berry *et al*, 2015; Weber & Brangwynne, 2015; Feric *et al*, 2016). However suggestive, these observations need to be reconciled with a considerably more variegated picture. Nucleolar assembly is a complex regulated process involving several hundred components, spatial cues (e.g., the position of nucleolar organizer regions, or NORs, on chromosomes), and energy expenditures at various steps (Savino *et al*, 2001; Shav‐Tal *et al*, 2005; Boisvert *et al*, 2007, 2010). Recent super‐resolution fluorescence microscopy analyses of fibrillarin and other components of the dense fibrillar component, as well as of the rDNA and associated proteins, demonstrated a considerably more complex substructure than predicted by PS experiments *in vitro* (Yao *et al*, 2019; Maiser *et al*, 2020).

Recently, NPM1, shown to have a fixed *C*_sat_ in isolation *in vitro*, was found not to have a fixed *C*_sat_ when its concentration was progressively increased within intact cells (none of several additional putative PS drivers of other nuclear bodies had one either) (Riback *et al*, 2020). When mixed *in vitro* with rRNA and the nucleolar protein SURF6, again no fixed *C*_sat_ was observed. Like many other nucleolar proteins, SURF6 contains a highly specific nucleolar localization signal (NoLS), known to bind NPM1 in multivalent configurations that can generate high binding affinities (Valdez *et al*, 1994; Kramer & Karpen, 1998; Mammen *et al*, 1998; Scott *et al*, 2010; Brabez *et al*, 2011; Mitrea *et al*, 2016). All evidence *in vitro* indicated that NPM1 and SURF6 bound each other, causing the ratio of NPM1 concentration in the dilute and dense phases to increase progressively as binding sites became saturated (Riback *et al*, 2020), a foregone expectation for a binding interaction.

In an undeterred new vision, the granular component of the nucleolus, in addition to the nucleolus itself, has been presented as a high‐dimensional condensate with multiple co‐influencing phases generated by *heterotypic* biomolecular interactions (Riback *et al*, 2020). Similar ideas were recently put forward for stress granules, another mainstay of general PS, after SSIs had re‐emerged there as the likely main drivers of assembly (Guillen‐Boixet *et al*, 2020; Sanders *et al*, 2020; Yang *et al*, 2020). While possibly formally correct, the description of compartments and parts thereof as high‐dimensional condensates is potentially deceiving: The new observations may not exclude PS, but do not implicate it either, while the complexity of this *ad hoc* description makes PS appear progressively less plausible. How would multidimensional condensates with hundreds of components with variable and co‐influencing *C*_sat_ look like and function? This is precisely the point when the analogy loses appeal and must be replaced with real molecular understanding.

## Special PS in the nuclear pore complex

Thus, general PS is a powerful analogy, but despite the flow of claims, evidence that it is the driver of biogenesis of any biological compartment of realistic complexity, a function for which it seems fundamentally inappropriate in comparison to SSIs, is thin, or probably even non‐existent. Better examples might eventually emerge, but will have to be scrutinized rigorously through shared criteria, with the established standards of research in molecular mechanistic biology that I presented above.

**Figure 6:**
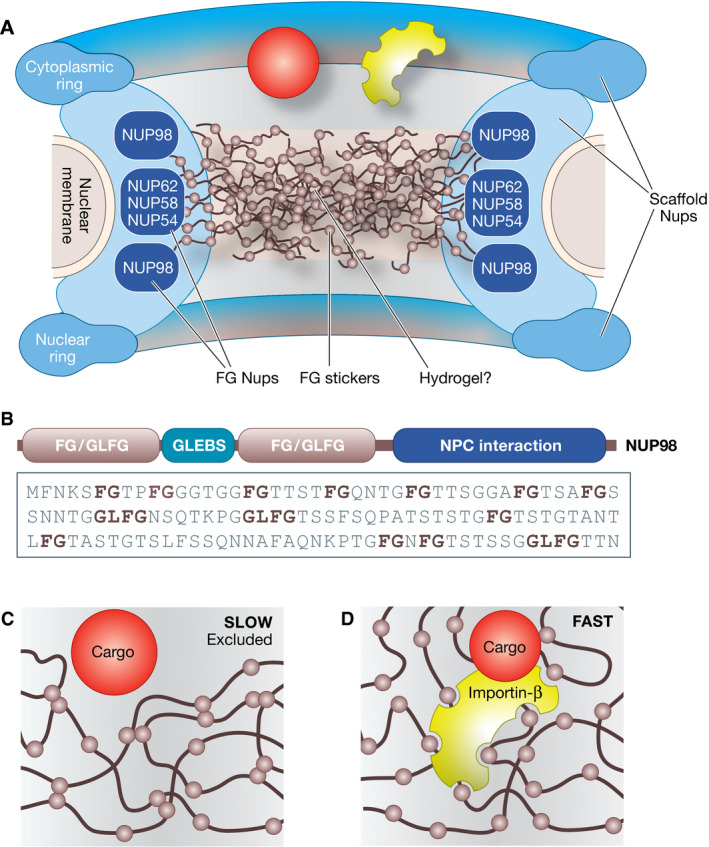
PS in the cavity of the nuclear pore complex (A) The Scaffold‐Nups subunits of the NPC (not shown individually) build a complex circular structure with a central cavity. The FG‐Nups (only a subset of which are shown) interact with the Scaffold‐Nups through SSIs. This allows to position their IDRs in the cavity of the pore, where they may form a hydrogel; (B) Domain organization of human NUP98, one of the human FG‐Nups. A short segment of the FG/GLFG sequence is shown; (C) An inert cargo molecule of sufficiently large size will be unable to cross the NPC cavity; (D) The cargo needs to bind to an import/export receptor like importin‐β, which binds the FG repeats, locally “melting” the meshwork, and therefore dissolving in it.

We should therefore discuss special PS, a “moderate” version of PS that assumes (i) that compartments are formed through SSIs, and (ii) that PS may manifest itself locally to meet a specific functional requirement. Although *in vitro* reconstitutions of phase‐separated compartments are also typical of studies of special PS, the motivation behind these experiments is different, as in special PS these experiments are not meant to identify putative drivers of compartment biogenesis. Rather, in special PS, these experiments are meant to test whether phase separation within the compartment can impart a specific functional feature. The best example involves the nuclear pore complex (NPC), a proteinaceous macromolecular complex that fuses the inner and outer nuclear membranes, generating a channel that crosses the nuclear envelope (Kabachinski & Schwartz, 2015; Hampoelz *et al*, 2019). The NPC assembles through SSIs of scaffold subunits, the scaffold nucleoporins (Scaffold‐Nups; Fig 6A), and appears as a complete membrane‐embedded ring with an inner cavity (Hampoelz *et al*, 2019).

The cavity is filled with the intrinsically disordered regions (IDRs) of a specialized subset of Nups, the FG‐Nups (Fig 6A). These contain large, low‐complexity disordered domains with characteristic phenylalanine glycine (FG) motifs and domains that bind the NPC scaffold via classical SSIs (Fig 6B) (Beck & Hurt, 2017). The FG‐Nups are required for the permeability barrier that controls material transfer between nucleoplasm and cytoplasm (Hurt, 1988; Wente *et al*, 1992; Patel *et al*, 2007; Hulsmann *et al*, 2012). Macromolecules with diameters above 4–5 nm or molecular masses above ~40 kDa fail to cross the NPC’s inner cavity with its ~50 nm diameter. Transport of these inert cargo macromolecules requires their interaction with a family of specialized nuclear transport receptors (NTRs) (Figs 1D and E, and 6C and D) (Bayliss *et al*, 1999; Gorlich & Kutay, 1999; Isgro & Schulten, 2005; Port *et al*, 2015). Thus, models of transport through the NPC need to explain what makes the majority of macromolecules inert and why the NTRs are selectively allowed to go through with their cargo (Frey *et al*, 2018).

The IDRs of FG‐Nups have an estimated concentration of 1 mM in the cavity of the NPC, and FG repeats may reach concentrations as high as 50 mM (Kabachinski & Schwartz, 2015; Schmidt & Gorlich, 2016; Beck & Hurt, 2017; Hampoelz *et al*, 2019). What material properties emerge from the clustering of the IDRs inside the NPC cavity? And how do these properties relate to the permeability barrier of the NPC? Several major competing hypotheses have emerged, covering the entire spectrum of possible phase behavior expected for IDRs (Lemke, 2016; Musser & Grunwald, 2016; Schmidt & Gorlich, 2016). In the virtual gate model, the IDRs of FG‐Nups, regarded as poorly cohesive, are proposed to align side by side, similarly to polyethylene glycol (PEG) molecules, to generate a “brush” that repels macromolecules but binds the NTRs, possibly coupled with a retraction mechanism upon NTR binding (Rout *et al*, 2003; Lim *et al*, 2007).

In the selective phase model (Ribbeck & Gorlich, 2001), on the other hand, the IDRs of a subset of cohesive FG‐Nups are proposed to undergo PS within the cavity of the NPC, driven by cohesive stacking and hydrophobic interactions of Phe residues in the FG repeats (Frey *et al*, 2006; Frey & Gorlich, 2007). Indeed, some of the most cohesive FG‐Nups stand out from a broader group, and more generally from IDPs, for a high level of hydrophobicity, comparable to that of folded globular proteins, and for almost negligible charge (no Arg, Lys, Asp, or Glu, and thus no repulsive forces) (Schmidt & Gorlich, 2015, 2016; Lemke, 2016). While PS of FG‐Nups does not occur outside the NPC without strong overexpression, FG hydrogels assemble *in vitro* from dilute solutions of FG‐Nups in aqueous buffers (Schmidt & Gorlich, 2015, 2016). Remarkably, these FG hydrogels recapitulate the main properties expected for the permselectivity barrier of the NPC, namely rejecting inert proteins of size larger than the hydrogel’s mesh size, and being a highly selective solvent for the NTRs (Frey *et al*, 2006; Frey & Gorlich, 2007).

The function of the NTRs is enabled by their being highly selective FG‐motif binders (Figs 1D and E, and 6C and D) (Iovine *et al*, 1995; Paschal & Gerace, 1995; Radu *et al*, 1995; Rexach & Blobel, 1995). Accordingly, NTRs are strikingly enriched on phenyl sepharose under highly stringent conditions (Ribbeck & Gorlich, 2002). Their navigation through the FG hydrogel is enabled by the ultrafast kinetics of their interactions with FG repeats, which involves multiple (probably up to eight or nine) FG‐binding sites on each NTR (Ribbeck & Gorlich, 2001; Isgro & Schulten, 2005; Frey & Gorlich, 2007; Milles *et al*, 2015; Port *et al*, 2015; Lemke, 2016). This allows NTRs and their associated cargo to cross the barrier within a few milliseconds (Yang *et al*, 2004), even if the overall binding affinity for FG motifs over multiple binding site on NTRs may be considerable. Thus, when the NTRs penetrate the mesh, they may temporarily dissolve the dense FG network (Schmidt & Gorlich, 2016).

The introduction of hexanediols, aliphatic alcohols, as tools to interfere with PS reflects the early ingenuous intuition that they might interfere specifically with the hydrophobic interactions between FG‐Nups and between FG‐Nups and NTRs, and thus interfere with hydrogel assembly and nuclear transport (Ribbeck & Gorlich, 2002). This approach had been developed in an age of innocence, but it is now becoming recognized that hexanediols have various negative consequences on cell physiology (Wheeler *et al*, 2016; Kroschwald *et al*, 2017; Duster *et al*, 2021; Itoh *et al*, 2021; Ulianov *et al*, 2021).

In the absence of direct ways to image the FG meshwork, biophysical work to test the selective phase hypothesis has been mainly performed *in vitro*. Work so far has been limited to especially cohesive FG‐Nups (Schmidt & Gorlich, 2015), thus falling short yet of explaining how the interplay of all FG‐Nups influences phase behavior and its consequences for transport through the NPC. Furthermore, FG‐Nups are strongly glycosylated, at least in metazoans (Labokha *et al*, 2013). How this modification influences cohesiveness and permselectivity, however, is unclear (Kabachinski & Schwartz, 2015; Schmidt & Gorlich, 2016). Finally, the interplay between FG‐Nups and NTRs remains poorly explored. These objections notwithstanding two decades of work on the selective phase hypothesis have generated a very large body of evidence in its support. Plausibly, it is the most advanced and best supported model of how phase properties of IDPs may cause PS and influence the material properties and function of a subcellular compartment. Anticipatory as it appears to be, this work has been puzzlingly ignored by the vast majority of reviews on the PS topic.

## Frustrating complexity

**Figure 7:**
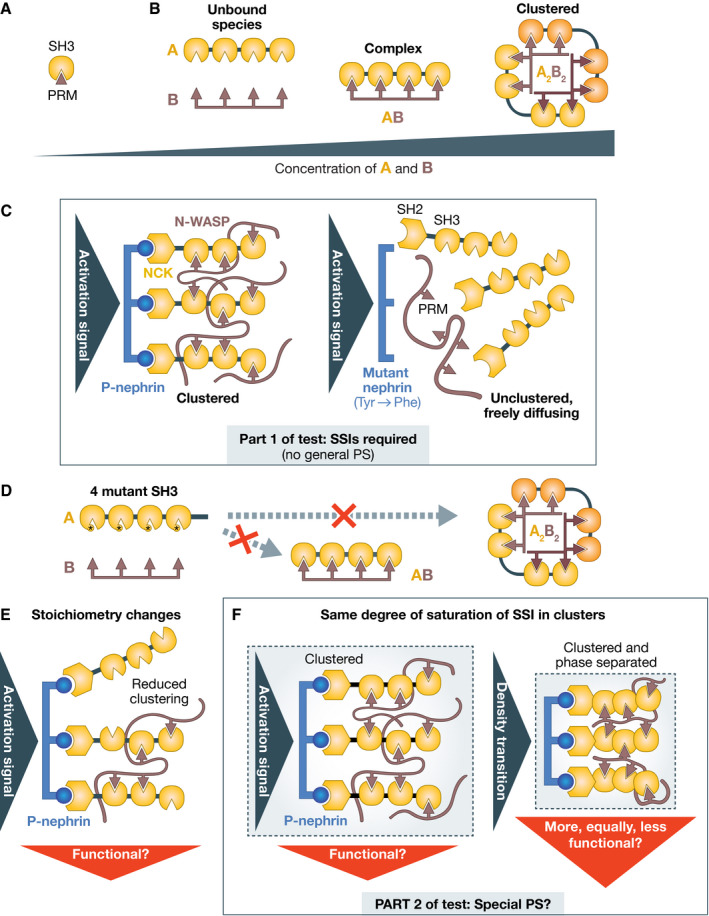
Complexity of a multivalent system (A) A single SH3/proline‐rich motif module may have relatively low affinity; (B) Multiple modules on the two A and B ligands bind each other more tightly. The complex forms first as the concentration of A and B is increased. Further increasing the concentration leads to assemble clustered complexes in which multiple multivalent ligands interact at the same time in a network. At high concentrations, this system undergoes PS *in vitro*, demonstrating that PS is perfectly compatible with the SSIs that this system is based on. (C) The nephrin/NCK/N‐WASP system does not configure general PS because the SSIs elicited by phosphorylation of nephrin at three different sites are required for recruiting downstream components and membrane clustering. Clustering occurs when bound NCK promotes further recruitment of N‐WASP, which crosslinks the NCK molecules near P‐nephrin. Thus, binding interactions are required at all stages of assembly of this membrane‐bound signaling compartment; (D) Putative mutations at the SH3/PRM interface prevent formation of the simple AB complex as well as of the clustered A_2_B_2_ complex, indicating SSIs are necessary for both; (E) Changing stoichiometries in clustered compartments changes the degree of clustering, which may lead to graded functional responses. This strategy does not probe PS but rather binding saturation; (F) Part 2 of the test aims to detect PS within a compartment held together by SSIs. Ideally, PS should be measured under conditions of equal saturation of the binding interactions between all components. The solubility‐determining linkers between interacting “stickers” may determine whether a density transition has taken place or not. Functional output should be compared for the two depicted scenarios.

The discussion of the selective phase hypothesis demonstrates that proving PS, even special PS, is enormously demanding. A discussion of multivalent systems further exemplifies this complexity. In Fig 7A and B, A and B are artificial MVPs containing multiple SH3 domains and PRMs (Li *et al*, 2012; Banani *et al*, 2016). In comparison to the interaction of individual SH3 and PRM modules (Fig 7A), the AB complex, with four interacting modules, can be expected to have very high affinity (Jencks, 1981; Mammen *et al*, 1998; Li *et al*, 2012). With concentrations of A and B increasing, the n stoichiometric AB complexes begin to interact *in trans* and cluster in distributed A_n_B_n_ configurations (Li *et al*, 2012) (a simple A_2_B_2_ is shown in Fig 7B). This may drive PS (see below).

In the biological context that inspired the construction of A and B, nephrin, a transmembrane receptor activated by external ligands, causes phosphorylation‐dependent binding to the NCK SH2 domain and membrane localization of NCK and N‐WASP (broadly equivalent to A and B, respectively, as shown in Fig 1F). Nephrin is also multivalent (Fig 7C), as it displays three phosphorylation sites for NCK (Jones *et al*, 2006; Blasutig *et al*, 2008). It is an integral agent of the clustering mechanism shown schematically in Fig 7C (Case *et al*, 2019). NCK and N‐WASP are therefore binding *clients* of nephrin (Fig 3). After recruitment to nephrin, they turn into secondary binding scaffolds themselves and recruit proteins involved in actin polymerization (Ditlev *et al*, 2012; Su *et al*, 2016; Case *et al*, 2019). Thus, NCK and N‐WASP are not general PS scaffolds as the experiments with A and B might have seemed to imply (Li *et al*, 2012). If they were, their clustering would trigger (undesirable) signaling independently from upstream signaling events.

As shown in Fig 7C and D, mutations of the interfaces that mediate SSIs in the AB complex or in the nephrin/NCK/N‐WASP system would prevent all steps of complex assembly and clustering. PS *in vitro* in these systems (Li *et al*, 2012; Case *et al*, 2019) depends on the ability of the interacting regions of MVPs to form clustered arrangements. This demonstrates, beyond reasonable doubt and in addition to arguments that I raised previously, that SSIs are perfectly compatible with PS and liquid‐like behavior, at least *in vitro*. This mutational analysis, which corresponds to Part 1 of the test as defined above, also implies that this is not a case of general PS.

How could we test if this system undergoes special PS *in vivo*? A reasonable initial assumption is that the sparse distribution of phospho‐nephrin will drive formation of multivalent clusters of finite size, appearing as small dots in a fluorescence microscope (Case *et al*, 2019). In elegant experiments, it was shown that changes in the relative stoichiometries of the multivalent nephrin/NCK/N‐WASP ligands in membrane clusters affected downstream events, such as the rate of assembly of actin filaments by the Arp2/3 complex (Case *et al*, 2019). Changes in stoichiometry, however, may simply modulate the level of binding saturation of the interacting domains in the cluster, which in turn is expected to modulate downstream signaling events. They do not imply PS and in fact may appear to counter the idea of an underlying *C*_sat_ (Fig 7E). Rather, because PS implies differential solvation and a density transition inside the compartment (Harmon *et al*, 2017), it should be tested under conditions in which the degree of saturation of binding interactions in the network remains *approximately constant*, as in the left and right panels of Fig 7F. Thus, despite a mention of PS in the title of the study (Case *et al*, 2019), whether this system undergoes PS *in vivo* is uncertain. Rather, we will need to distinguish between simple multivalent clusters and condensed, phase‐separated clusters (Fig 7F). How can this be approached?

In an initial theoretical treatment of PS in the simpler AB model system depicted in Fig 7B, PS was proposed to occur when the gain in configurational entropy compensates for the loss of translational and rotational entropy of the entities undergoing PS (Li *et al*, 2012). Later, this was partly amended and the increase in configurational entropy was rather proposed to drive clustering. In computational models, clustering might take the form of physical systems‐wide gelation (without PS) or of gelation driven by PS, the latter promoted by the specifics of the often long and disordered linkers between interacting domains (Banjade *et al*, 2015; Harmon *et al*, 2017). Even merely intuitively, focus on linkers is sensible because compartments lack long‐range order, and intervening flexible linkers allow domains to orient themselves freely for interactions with cognate modules. Conformational changes in the linkers may drive a more compact and dense organization.

In a real system like that in Fig 7F, clusters, as discussed above, are necessarily of finite size due to the overall small number of molecules available. Distinguishing between local gelation and local gelation driven by PS in these clusters will require manipulating the sequence of the interdomain linkers predicted to be the crucial solubility determinants (Banjade *et al*, 2015; Harmon *et al*, 2017; Choi *et al*, 2020), while measuring functional consequences of these manipulations ideally together with measurements of the density of the clusters (as it may be revealed by super‐resolution microscopy methods, for instance). However, in preparing for these experiments, there is a sobering gap between knowns and unknowns. We may have a theory for how the length and sequence of the interdomain linkers influence solubility in a defined buffer (Harmon *et al*, 2017; Choi *et al*, 2020), but we do not know how this will influence the interaction of the domain modules that the linkers separate. Model multivalent systems of identical motifs like AB may promote increases in configurational entropy more efficiently than systems made of more selectively interacting pairs, with a more restricted number of available configurations, the achievement of which may critically depend on linker length. Thus, besides solvation, linkers may also influence possible geometries restricting or favoring clustering. Linkers have rapidly diverging sequences, which complicates the formulation of simple, testable mechanistic hypotheses (van der Lee *et al*, 2014). Furthermore, we may not know many of the factors that determine solubility in the cellular environment, and should anticipate unexpected outcomes because of this. Thus, there are many thermodynamic variables in these experiments whose effects are difficult to control.

## PS should explain something

Proving that PS exists and that it is functionally relevant, even in this second relatively simple system of purported special PS, will be an extremely difficult and tedious task. An examination of Fig 7F clarifies that there is no obvious reason why a simple cluster (left) should be any less functionally active than a phase‐separated cluster (right). In fact, the opposite may be true because a dense cluster may be expected to slow diffusion of effectors into the clusters. Thus, in this case, it is more difficult to grasp, in comparison to the NPC case, what functional need PS would address that could not be addressed without it.

The same concern can be expressed for the purported PS of the chromosomal passenger complex (CPC) (Trivedi *et al*, 2019). The CPC is a validated client of phosphorylation signals on centromeric chromatin (Fig 3) (Kelly *et al*, 2010; Wang *et al*, 2010; Yamagishi *et al*, 2010), but like various other examples, it was recast as an *in vitro* PS scaffold and proposed to phase‐separate after it reaches high concentrations at the centromere (Trivedi *et al*, 2019), with unclear functional gains. Neither of three related crucial tests in support of PS of the CPC was performed (Trivedi *et al*, 2019). First, it is unknown whether the cytosolic concentration of the CPC remains constant (*C*_sat_) during its accumulation at the centromere, a necessary condition to claim PS. Second, if PS was nucleated by concentration of the CPC at the centromere as proposed, PS should persist after removal of the initial spatial cues, in this case the phosphorylation signals that attract it to the centromere [see discussion in (Erdel & Rippe, 2018)]. Third, after nucleation of PS, additional CPC will be expected to be recruited independently of any spatial cues, including mutants unable to bind the phosphorylation cues [and independently of dimerization with the wild‐type complex (Bourhis *et al*, 2009; Bonner *et al*, 2020)].

Special PS may play a role in RNP granules (Protter *et al*, 2018), as well as on the surface of chromosomes, as suggested, for instance, by studies on Ki67, a surfactant that promotes the individualization of chromosomes during mitosis (Cuylen *et al*, 2016; Cuylen‐Haering *et al*, 2020). Chromosomes themselves, with a high local concentration of differently modified nucleosomes, may also be a favorable terrain for special PS. However, the technical and conceptual difficulties of studying special PS in this context are well exemplified by the controversy on the role of HP1 in the organization of heterochromatin (Larson *et al*, 2017; Strom *et al*, 2017; Sanulli *et al*, 2019; Erdel *et al*, 2020).

## Conclusions

The study of PS in biology has grown explosively, sparking new and important research in polymer chemistry, material science, synthetic biology, origin of life, and the pathology of aggregation [e.g., (Jawerth *et al*, 2020; Reinkemeier & Lemke, 2021; Te Brinke *et al*, 2018; Wei *et al*, 2017)], often displaying an impressive degree of technical and intellectual sophistication. Part of the enormous success of the PS idea is driven by the legitimate anticipation that it will uncover new mechanisms in the area of neurodegenerative diseases and aggregative phenomena. Here, I exclusively focused on the molecular mechanisms of compartment assembly and on the potential role of PS in biology. I clarified that the apparent simplicity of the PS hypothesis is deceiving, as the general PS idea is based on rather implausible assumptions. Implausible does not imply erroneous. Nonetheless, the arguments presented here provide compelling evidence that the majority of PS claims have been based on highly incomplete tests that ignored more plausible drivers of macromolecular concentration. This criticism applies to the vast majority of PS claims in the literature, as can be easily verified retrospectively. Focusing on the mechanism of compartment assembly, my analysis echoes and complements a previous critique that questioned PS based on the appearance of compartments (McSwiggen *et al*, 2019b).

The study of the specificity of binding interactions builds on decades of tedious analyses that unveiled specificity rules and described them on the basis of their biochemistry, often providing very satisfactory explanations of the effects of mutation of (usually) conserved residues on phenotype. One should therefore provide very strong reasons in order to replace, or bypass, this framework with a new one, and any new framework should be expected to explain issues that were recalcitrant in the earlier view. Alternative hypotheses need to be considered and excluded (Part 1 and Part 2 of the test), but cannot be ignored. The colloidal science premises of the PS hypothesis were formulated in the first half of the twentieth century [see discussion in Hyman and Brangwynne (2011)], before the explosion of molecular biology, and ignorance of the crucial details made simplifications and analogies unavoidable back then. Nowadays, PS can only be a pillar of molecular mechanistic research.

The study of compartment biogenesis and organization should aim to reach the standards elucidated by work on the selective phase model (Ribbeck & Gorlich, 2001; Schmidt & Gorlich, 2016). The assembly principles of the NPC hydrogel are relatively simple in comparison to those of other cellular compartments. A major simplification is that NPC transport is thermodynamically driven, as it does not imply active processes within the NPC itself. Directionality of transport processes is ensured by gradients of the Ran GTPase outside the NPC (Schmidt & Gorlich, 2016). Compartments like the nucleolus show considerably greater complexity than the NPC. There is no simple pipeline to attack these structures and their (usually) non‐equilibrium dynamics. Modeling active processes *in vitro* with interacting parts capable of feedback control is a tremendous challenge that will project molecular mechanistic and synthetic biology into the future.

Even for simple compartments, there is more than a single laboratory can do, raising the question whether new models of cooperation are required. Progress will likely emerge from a combination of accurate part listings, complex reconstitutions, direct imaging (e.g., through cryo‐electron tomography and advanced super‐resolution fluorescence methods), simulation and theory, microfluidics, and more. The challenges ahead are substantial and so are the rewards, which include our future ability to understand and control biological objects and subcellular compartments of enormous complexity, including our chromosomes and the machinery that mediates intercellular communication.

## Supporting information


