# Supplementary material for: On the role of phase separation in the biogenesis of membraneless compartments
Source: EMBO J. 2022 Feb 2;41(5):e109952. doi: 10.15252/embj.2021109952 (PMC8886532; doi:10.15252/embj.2021109952)
Supplement: Supplementary file 1 [file EMBJ-41-e109952-s001.pdf]

THE EMBO JOURNAL

On the role of phase separation in the biogenesis of membraneless compartments

Andrea Musacchio
DOI: 10.15252/embj.2021109952

Corresponding author(s): Andrea Musacchio (andrea.musacchio@mpi-dortmund.mpg.de)

Review Timeline:

Submission Date:	14th Oct 21
Editorial Decision:	25th Nov 21
Revision Received:	7th Dec 21
Accepted:	10th Jan 22

Transaction Report:

(Note: With the exception of the correction of typographical or spelling errors that could be a source of ambiguity, letters and reports are not edited. Depending on transfer agreements, referee reports obtained elsewhere may or may not be included in this compilation. Referee reports are anonymous unless the Referee chooses to sign their reports.)

1st Editorial Decision — 25th Nov 2021

Thank you again for submitting your essay on membraneless compartments for our consideration. I have now received the below-copied comments from three expert referees, who all found the piece interesting, timely and informative. As you will see, referees 1 and 3 only have minor suggestions. Referee 2 finds that the clarity of a few passages might still be improved, so please carefully consider these comments and how they might be incorporated into a revised version of the piece. Addition of a glossary listing recurrently used abbreviations and defining key (technical) terms would in my view also be helpful.

Referee #1:

The potential of the concept of "phase separation" (PS) to explain several biological and pathological phenomena is currently an extremely hot and controversial topic. In this review, Musacchio casts a critical eye over much of the fields current thinking on how PS might apply to the formation of membraneless compartments. Mussachio is an experienced and knowledgeable biochemist, but he clearly has written this review with biologists in mind. As a biologist, I found this a compelling and entertaining read that explained well the underlying chemistry (at least to my biologists knowledge) and did an excellent job of highlighting some of the problems with the conclusions drawn from the plethora of experiments that are currently interpreted as supporting PS, and also explaining the many challenges in assessing these conclusions more rigorously. Personally, I have always been somewhat sceptical that PS neatly describes as much as is claimed, so I am receptive to these arguments. Nevertheless, this seems a well-balanced, but straight-talking, review that provides a welcome counter to the current groundswell of enthusiasm for PS in biology. Although several recent reviews have taken a similar sceptical tone, I believe Musacchio's has the potential to be one of the more influential, and one can see from the acknowledgements that he has taken great care to discuss and consult with many of the main players in the field. I therefore strongly support publication and have only a few minor points that the author should consider prior to publication.

1. I found some of the Figure legends were slightly confusing or needed a bit more information for readers to fully understand them. For example, Proline-rich-motif not defined as "PRM" in Figure 1; in Figure 2D, Q and Y are considered stickers, but I wasn't sure by which mechanism shown in Figure 2B these interact; In Figure 3, the legend refers to transcripts being required for Cajal body formation, but in the Figure transcripts, histones and spliceosomes appear to perform a similar function (histones and spliceosomes not mentioned in the legend). These are all relatively minor, but these little things can add up.

2. P4, para.1: "assay" should be "essay".

3. P8, para.2: I did not understand the meaning of the first sentence, specifically the reference to "binding to a prominent but limiting contaminant".

Referee #2:

Andrea Musacchio contrasts phase separation (PS) as a driver for the assembly of membraneless cellular compartments to classical binding models and stereospecific interactions (SSI) that involve a certain 3D fold of at least one interaction partner. He challenges the view that relatively weak unspecific interactions are the main determinants of cellular subcompartment assembly. The article makes some very good and thought-provoking comments on the PS paradigm. It takes a refreshing perspective at the question if liquid-liquid phase separation (LLPS) is indeed a relevant mechanism for structuring the cell under endogenous conditions. A broad range of aspects is covered, and the author backs up his claims with a quite comprehensive and appropriate set of references. Overall, it is a timely, thoughtful and, at times, provocative but much needed discussion of phase separation (PS) as a mechanism for cellular compartmentalization. By pointing out the need to define specific molecular features and implications of PS more clearly and compare them to classical SSI driven assembly mechanisms, it will certainly stimulate further discussions. Thus, it will be helpful to sharpen our view on biophysical principles that govern cellular compartmentalization. Below I have included some comments and questions that the author might want to consider.

1. Given the controversial nature of the topic and the pointed position taken by the author, I would not use the current article type classification as "Review". Designating it as a "Commentary", "Opinion" or "Essay" type of paper seems more appropriate. That is evident already from the title and the author refers to the article as an essay himself.

2. The classification into "general" PS with weak and unspecific interactions vs. "special" PS around a scaffold formed by SSI seems somewhat artificial to me. In my opinion, there is some consensus that the general "pure" PS designation as defined by Musacchio would only apply to in vitro PS with a limited number of purified proteins. Studies that invoke a PS mechanism for cellular compartment formation would usually not exclude at all that assembly by PS is targeted by nucleation sites formed by SSIs. A case in point is the nucleolus. The PS mechanism as reviewed in Lafontaine 2021 (PMID: 32873929) would explicitly include rDNA sequences and SSIs nucleation sites. It proposes a surface condensation type of PS and does not claim that the nucleolus is formed by pure LLPS without SSIs. As the author points out, the same would apply for the centromere/CENP-A or the nuclear pore as a prototypic case of special PS. Thus, to me, the more interesting question would be whether PS makes a functionally relevant contribution to the formation/maintenance of a cellular compartment.

3. The distinction between specific/high affinity (= no PS) vs. unspecific/weak affinity (= PS) seems questionable to me. Apart from the challenge to decide what is weak/strong or specific/unspecific, the formal requirement for PS would be that multi-valent interactions are present. Depending on their affinity, the type of resulting PS driven compartment could be liquid-, gel- or solid-like. Thus, multi-valent specific/higher affinity interactions could also be drivers of a PS mechanism.

4. Importantly, and in contrast to a variety of recent studies and reviews, the comment makes a very good effort to clearly define the relevant terms. It should be checked that this is complete with respect to what is needed for the general reader of EMBO J. For example, when the term "saturating concentration (Csat)" is used first at the bottom of p. 3 it should be clarified that at concentrations below Csat, the system is in the one-phase regime. A glossary of key terms and their abbreviations would be useful, too.

Sections "Validation pipeline part 1/2/shortcuts", general comment: These sections addresses the important issue on how to distinguish between different mechanisms. In its present form, I do not find it particularly useful with respect to suggesting specific experiments that are informative. The three sections seem to be mostly focused on describing the complexity of the issues and listing challenges, caveats, and unanswered questions. While the points raised are well taken, they do not fit well to the "validation pipeline" title as well as the initial question "How should we investigate this question [if PS is a driver] for other compartments where our knowledge of the assembly mechanisms is limited?" Strengthening this part would increase the value of the essay. Some specific issues for these sections are described below.

5.

6. Validation pipeline part 1, FRAP: As pointed out previously (McSwiggen 2019b) and in the essay, the absolute value of koff of a given factor measured by FRAP is indeed not suited to conclude whether a compartment is formed by phase separation or not. However, measuring protein transport within the compartment (internal mixing) versus exchange in and out of it from the cellular environment provides valuable information to assess whether a LLPS mechanism is present (Brangwynne 2009, Erdel 2020). Thus, I do not find the argument convincing that FRAP measurements would be useless because recovery of different factors occurs on different time scales.

7. Validation pipeline part 1, compartment composition and specificity/hierarchy of interactions: Mapping the composition of a given compartment is certainly a valuable part of its characterization. However, the mechanistic insight gained from this is limited. Using again the nucleolus as an example: Its protein, RNA and DNA content have been comprehensively mapped, but what does this mean with respect to whether PS is relevant or not for its assembly? Likewise, trying to exclude SSIs seems like a futile exercise to me ("If they [SSIs] had not been found, compartment X might qualify for general PS", see also comments above). It is actually very, very difficult to prove that something is not existing...

8. Validation pipeline: part 2/shortcuts: I have problems to understand these two parts and some clarifications might be helpful. First, it is not clear to me, why "part 2" and "shortcuts" are separated as both seem address the same issues. They both discuss perturbance experiments and the difficulties to determine Csat in the cell. After raising the latter point, "part 2" ends with a long list of questions without answers that do not help me to understand how the validation pipeline would/should work. In the "shortcuts" part it mostly seems to say that perturbing a putative PS driver in the cell is not a good approach because it might not be possible to measure Csat. I would argue that it is nevertheless a very informative experiment to perturb the concentration of a given potential PS driver in the cell and then evaluate how this affects the structure of a given compartment. If you raise the concentration of factor X and the compartment does not increase in size or its structure is disrupted, this argues against LLPS being involved. In addition, the lack of a knock-down/knock-out structure phenotype would indicate that the corresponding factor is unlikely to be a PS driver although it might form liquid droplets in vitro.

Referee #3:

In the manuscript entitled 'Membraneless compartments: Doctoring the vinaigrette with specificity', Andrea Musacchio lays out a cogent essay arguing that site specific interactions, with known and established physicochemical properties (site specific interactions, on and off rates), provide plausible and more realistic models for most condensate/phase separation phenomena, an argument that is largely contrary to models that rely almost solely on weak non-specific interactions derived from in vitro data or somewhat simplistic interpretation of in vivo experiments. Instead of tackling every area of biology that has jumped on condensates as an explanation for subcellular organization, Musacchio wisely focuses on a few areas of biology where some form of phase separation may really occur (NPC for instance), but only after architectures are established by specific interactions. Musacchio argues successfully, in my opinion, that many phenomena attributed to condensates in vivo (via FRAP) or in vitro fusing of condensates could easily, and perhaps more readily be explained by high and low affinity specific interactions coupled with fast or slow off rates, a feature of the physical universe that many have forgotten in their search for new biology. It is imperative that articles like this be published as they serve to correct a tendency to reward new models too enthusiastically. In this case, by explaining why phase separation need not be invoked in most cases to explain the physical phenomena of membraneless compartments. Furthermore, Musacchio lays out strategies to test hypotheses, and he helps define systems that deserve more attention given their clear biological roles in presenting phase barriers between the nucleus and cytoplasm (NPC).

Since it appears clear that Musacchio consulted many scientists on all sides of condensate biology (see acknowledgements), I am hopeful that most found his arguments useful with respect to testing hypotheses related to phase separation/condensates. I hope this to be true, but those scientists who might benefit most from reading this piece might also be put off by two parts in the intro regarding definitions of condensates and stereospecific interactions. I completely agree with the author, these terms are inappropriately coopted and misused, but I seriously doubt that other authors will stop using them anytime soon, and it seemed to me that those paragraphs were written in a somewhat patronizing tone. Perhaps Musacchio could simply define his new and more accurate terminology by stating that prior terms are inadequate.

Minor typos:

Page 1 'even hundreds different' should be 'even hundreds of different'

First line of page 4, 'In this assay' should be 'In this essay'

Bottom of page 7, 'the questions how solubility' should maybe be 'the questions of how solubility'

Referee #1:

The potential of the concept of "phase separation" (PS) to explain several biological and pathological phenomena is currently an extremely hot and controversial topic. In this review, Musacchio casts a critical eye over much of the fields current thinking on how PS might apply to the formation of membraneless compartments. Mussachio is an experienced and knowledgeable biochemist, but he clearly has written this review with biologists in mind. As a biologist, I found this a compelling and entertaining read that explained well the underlying chemistry (at least to my biologists knowledge) and did an excellent job of highlighting some of the problems with the conclusions drawn from the plethora of experiments that are currently interpreted as supporting PS, and also explaining the many challenges in assessing these conclusions more rigorously. Personally, I have always been somewhat sceptical that PS neatly describes as much as is claimed, so I am receptive to these arguments. Nevertheless, this seems a well-balanced, but straight-talking, review that provides a welcome counter to the current groundswell of enthusiasm for PS in biology. Although several recent reviews have taken a similar sceptical tone, I believe Musacchio's has the potential to be one of the more influential, and one can see from the acknowledgements that he has taken great care to discuss and consult with many of the main players in the field. I therefore strongly support publication and have only a few minor points that the author should consider prior to publication.

I am very grateful to the reviewer for a positive assessment of the essay.

1. I found some of the Figure legends were slightly confusing or needed a bit more information for readers to fully understand them. For example:

I apologize for the shortcomings and I tried to address this point.

Proline-rich-motif not defined as "PRM" in Figure 1;

The definition has been added

In Figure 2D, Q and Y are considered stickers, but I wasn't sure by which mechanism shown in Figure 2B these interact

I have added a clarification to the legend

In Figure 3, the legend refers to transcripts being required for Cajal body formation, but in the Figure transcripts, histones and spliceosomes appear to perform a similar function (histones and spliceosomes not mentioned in the legend).

Also in this case, a clarification has now been added to the legend.

These are all relatively minor, but these little things can add up.

2. 4, para.1: "assay" should be "essay".

Corrected

3. P8, para.2: I did not understand the meaning of the first sentence, specifically the reference to "binding to a prominent but limiting contaminant".

I agree that the original sentence was cryptic. I have reformulated it to make my thoughts more accessible.

Referee #2:

Andrea Musacchio contrasts phase separation (PS) as a driver for the assembly of membraneless cellular compartments to classical binding models and stereospecific interactions (SSI) that involve a certain 3D fold of at least one interaction partner. He challenges the view that relatively weak unspecific interactions are the

main determinants of cellular subcompartment assembly. The article makes some very good and thought-provoking comments on the PS paradigm. It takes a refreshing perspective at the question if liquid-liquid phase separation (LLPS) is indeed a relevant mechanism for structuring the cell under endogenous conditions. A broad range of aspects is covered, and the author backs up his claims with a quite comprehensive and appropriate set of references. Overall, it is a timely, thoughtful and, at times, provocative but much needed discussion of phase separation (PS) as a mechanism for cellular compartmentalization. By pointing out the need to define specific molecular features and implications of PS more clearly and compare them to classical SSI driven assembly mechanisms, it will certainly stimulate further discussions. Thus, it will be helpful to sharpen our view on biophysical principles that govern cellular compartmentalization. Below I have included some comments and questions that the author might want to consider.

I am very grateful to the reviewer for supporting publication of the essay and for making several valuable suggestions, which are addressed below and in a revised version of the manuscript.

1. Given the controversial nature of the topic and the pointed position taken by the author, I would not use the current article type classification as "Review". Designating it as a "Commentary", "Opinion" or "Essay" type of paper seems more appropriate. That is evident already from the title and the author refers to the article as an essay himself.

I agree with the reviewer. This was not meant as a review article, but precisely as Commentary, Opinion or Essay.

2. The classification into "general" PS with weak and unspecific interactions vs. "special" PS around a scaffold formed by SSI seems somewhat artificial to me. In my opinion, there is some consensus that the general "pure" PS designation as defined by Musacchio would only apply to in vitro PS with a limited number of purified proteins. Studies that invoke a PS mechanism for cellular compartment formation would usually not exclude at all that assembly by PS is targeted by nucleation sites formed by SSIs. A case in point is the nucleolus. The PS mechanism as reviewed in Lafontaine 2021 (PMID: 32873929) would explicitly include rDNA sequences and SSIs nucleation sites. It proposes a surface condensation type of PS and does not claim that the nucleolus is formed by pure LLPS without SSIs. As the author points out, the same would apply for the centromere/CENP-A or the nuclear pore as a prototypic case of special PS. Thus, to me, the more interesting question would be whether PS makes a functionally relevant contribution to the formation/maintenance of a cellular compartment.

I respectfully disagree with the reviewer on this point. What I tried to point out in my essay is that the field has proposed an inverse set of values that identified phase separation as the driving force of compartment assembly, and specific interactions as a corollary. This is testified by a flurry of papers where "Phase separations drives…" was the main message of the title or the abstract. These papers were all supported by insignificant *in vitro* experiments with purified proteins that were said to be predictive of PS in vivo. I think it remains important for the general audience to be given arguments for why these experiments were, for the most part, not predictive. Specifically, Lafontaine *et al.* 2021 came to introduce the multiphase idea after they finally realized (Ribeck et al. 2020) that the simple idea of PS was untenable. My personal opinion is that Ribeck et al. represents the lowest point of this saga.

3. The distinction between specific/high affinity (= no PS) vs. unspecific/weak affinity (= PS) seems questionable to me. Apart from the challenge to decide what is weak/strong or specific/unspecific, the formal requirement for PS would be that multi-valent interactions are present. Depending on their affinity, the type of resulting PS driven compartment could be liquid-, gel- or solid-like. Thus, multi-valent specific/higher affinity interactions could also be drivers of a PS mechanism.

I thank the reviewer for raising this point. I would like to note that the essay addresses the distinction precisely as pointed out by the reviewer. In the section titled *Macromolecular Interactions*, I write "Thus, while in principle both type I and type II/III interactions can reach high affinity (*e.g.*, for type I interactions by harnessing multivalency), the hallmark that distinguishes I from II and III is the low specificity of the first and the high specificity of the latter two." In a subsequent section titled *Frustrating complexity* I point out that PS in a multivalent system after translocation of system components to a membrane is plausible, but that demonstrating it is, for various reasons, an exceptionally complex endeavor. I also argued that experiments of titration of a binding species seem insufficient, if not inappropriate, to address the PS question, as by definition in PS systems the concentration of species inside or outside the compartment should not change as a function

of added interactors. I should also add that PS has now been claimed for a variety of non-multivalent systems, and that therefore the proponents of PS have themselves abandoned that requirement.

4. Importantly, and in contrast to a variety of recent studies and reviews, the comment makes a very good effort to clearly define the relevant terms. It should be checked that this is complete with respect to what is needed for the general reader of EMBO J. For example, when the term "saturating concentration (Csat)" is used first at the bottom of p. 3 it should be clarified that at concentrations below Csat, the system is in the one-phase regime. A glossary of key terms and their abbreviations would be useful, too.

Thank you, I have now included a reference to the one-phase regime. With concern to the glossary, I am torn. A certain vagueness and liberality of definitions has helped the field flourish, while this essay is an incitement to be rigorous. I tried to offer readers many criteria, so far ignored, that would help them assess the plausibility of considering PS as a driver in compartment assembly. What I would not want to happen is that attention is diverted to accuracy of my definitions in the glossary and for this reason I feel I'd rather avoid writing one.

5. Sections "Validation pipeline part 1/2/shortcuts", general comment: These sections addresses the important issue on how to distinguish between different mechanisms. In its present form, I do not find it particularly useful with respect to suggesting specific experiments that are informative. The three sections seem to be mostly focused on describing the complexity of the issues and listing challenges, caveats, and unanswered questions. While the points raised are well taken, they do not fit well to the "validation pipeline" title as well as the initial question "How should we investigate this question [if PS is a driver] for other compartments where our knowledge of the assembly mechanisms is limited?" Strengthening this part would increase the value of the essay. Some specific issues for these sections are described below.

I thank the reviewer for raising these concerns, which are answered specifically below.

6. Validation pipeline part 1, FRAP: As pointed out previously (McSwiggen 2019b) and in the essay, the absolute value of koff of a given factor measured by FRAP is indeed not suited to conclude whether a compartment is formed by phase separation or not. However, measuring protein transport within the compartment (internal mixing) versus exchange in and out of it from the cellular environment provides valuable information to assess whether a LLPS mechanism is present (Brangwynne 2009, Erdel 2020). Thus, I do not find the argument convincing that FRAP measurements would be useless because recovery of different factors occurs on different time scales.

The reviewer is correct and I apologize if I gave that impression. I have delegated much of the discussion on the subtlety of FRAP experiments to competent recent reviews. As I agree with the reviewer, I have now included a sentence that refers more explicitly to more complex options for FRAP analysis and added the Erdel *et al.* 2000 reference as well as a different and more recent reference from the Brangwynne laboratory that deals with FRAP specifically (Taylor et al. 2019).

7. Validation pipeline part 1, compartment composition and specificity/hierarchy of interactions: Mapping the composition of a given compartment is certainly a valuable part of its characterization. However, the mechanistic insight gained from this is limited. Using again the nucleolus as an example: Its protein, RNA and DNA content have been comprehensively mapped, but what does this mean with respect to whether PS is relevant or not for its assembly? Likewise, trying to exclude SSIs seems like a futile exercise to me ("If they [SSIs] had not been found, compartment X might qualify for general PS", see also comments above). It is actually very, very difficult to prove that something is not existing...

I agree with the reviewer that it is very difficult to prove that something is not existing, which is also the reason why claims of PS have been almost invariably premature. Just to take the nucleolus as an example, the interactions motifs that determine the localization of nucleolar proteins were known since 2006. A more productive course of action, rather than beginning with a claim of phase separation, later evolving into multiphase separation, was to ask if a *Csat* can be measured at all when binding partners of the "drivers" are present.

8. Validation pipeline: part 2/shortcuts: I have problems to understand these two parts and some clarifications might be helpful. First, it is not clear to me, why "part 2" and "shortcuts" are separated as both seem address the same issues. They both discuss perturbance experiments and the difficulties to determine Csat in the cell.

After raising the latter point, "part 2" ends with a long list of questions without answers that do not help me to understand how the validation pipeline would/should work. In the "shortcuts" part it mostly seems to say that perturbing a putative PS driver in the cell is not a good approach because it might not be possible to measure Csat. I would argue that it is nevertheless a very informative experiment to perturb the concentration of a given potential PS driver in the cell and then evaluate how this affects the structure of a given compartment. If you raise the concentration of factor X and the compartment does not increase in size or its structure is disrupted, this argues against LLPS being involved. In addition, the lack of a knock-down/knock-out structure phenotype would indicate that the corresponding factor is unlikely to be a PS driver although it might form liquid droplets in vitro.

In the original version of the manuscript, Part 2 and Shortcuts identified respectively what questions a rigorous PS test would entail and how the test is usually being run. I have now merged the two sections. The first part of the new section enumerates, in form of questions, what one should try to know to begin to distinguish PS from other mechanisms of biogenesis. In the second part, I indicate that the vast majority of studies has never even addressed the question whether a C*sat* can be measured at all. In their 2020 Nature paper, Riback *et al.* finally measured it for nucleolar components previously said, by the same authors, to phase separate. A fixed C*sat* could not be identified. Instead of admitting the problem, Riback *et al.* claimed multiphase behavior in sub-nucleolar compartments, something that I explicitly criticize in a subsequent section (the multiphase dilution of PS).

Referee #3:

In the manuscript entitled 'Membraneless compartments: Doctoring the vinaigrette with specificity', Andrea Musacchio lays out a cogent essay arguing that site specific interactions, with known and established physicochemical properties (site specific interactions, on and off rates), provide plausible and more realistic models for most condensate/phase separation phenomena, an argument that is largely contrary to models that rely almost solely on weak non-specific interactions derived from in vitro data or somewhat simplistic interpretation of in vivo experiments. Instead of tackling every area of biology that has jumped on condensates as an explanation for subcellular organization, Musacchio wisely focuses on a few areas of biology where some form of phase separation may really occur (NPC for instance), but only after architectures are established by specific interactions. Musacchio argues successfully, in my opinion, that many phenomena attributed to condensates in vivo (via FRAP) or in vitro fusing of condensates could easily, and perhaps more readily be explained by high and low affinity specific interactions coupled with fast or slow off rates, a feature of the physical universe that many have forgotten in their search for new biology. It is imperative that articles like this be published as they serve to correct a tendency to reward new models too enthusiastically. In this case, by explaining why phase separation need not be invoked in most cases to explain the physical phenomena of membraneless compartments. Furthermore, Musacchio lays out strategies to test hypotheses, and he helps define systems that deserve more attention given their clear biological roles in presenting phase barriers between the nucleus and cytoplasm (NPC).

Since it appears clear that Musacchio consulted many scientists on all sides of condensate biology (see acknowledgements), I am hopeful that most found his arguments useful with respect to testing hypotheses related to phase separation/condensates. I hope this to be true, but those scientists who might benefit most from reading this piece might also be put off by two parts in the intro regarding definitions of condensates and stereospecific interactions. I completely agree with the author, these terms are inappropriately coopted and misused, but I seriously doubt that other authors will stop using them anytime soon, and it seemed to me that those paragraphs were written in a somewhat patronizing tone. Perhaps Musacchio could simply define his new and more accurate terminology by stating that prior terms are inadequate.

I am very grateful to the reviewer for a positive assessment of the essay. I am also grateful for the suggestion to revise the discussion of terms used too liberally in the PS community, which I have tried to address in a revised version of the manuscript. I hope the relevant section will come across as less patronizing after this revision.

Minor typos:

Page 1 'even hundreds different' should be 'even hundreds of different'

Corrected

First line of page 4, 'In this assay' should be 'In this essay'

Corrected

Bottom of page 7, 'the questions how solubility' should maybe be 'the questions of how solubility'

Modified as suggested
